# Development of Highly Selective Kv1.3-Blocking Peptides Based on the Sea Anemone Peptide ShK

**DOI:** 10.3390/md13010529

**Published:** 2015-01-16

**Authors:** Michael W. Pennington, Shih Chieh Chang, Satendra Chauhan, Redwan Huq, Rajeev B. Tajhya, Sandeep Chhabra, Raymond S. Norton, Christine Beeton

**Affiliations:** 1 Peptides International Inc., 11621 Electron Drive, Louisville, KY 40065, USA; E-Mail: schauhan@pepnet.com; 2 Medicinal Chemistry, Monash Institute of Pharmaceutical Sciences, Monash University, Parkville, VIC 3052, Australia; E-Mails: jchangsc@gmail.com (S.C.C.); sandeep.chhabra@monash.edu (S.C.); ray.norton@monash.edu (R.S.N.); 3 Department of Molecular Physiology and Biophysics, Baylor College of Medicine, One Baylor Plaza, Houston, TX 77030, USA; E-Mails: ruhuq@bcm.edu (R.H.); tajhya@bcm.edu (R.B.T.); beeton@bcm.edu (C.B.)

**Keywords:** immunomodulator, T lymphocyte, potassium channel, disulfide-rich peptide, sea anemone toxin, K^+^ channel blocker

## Abstract

ShK, from the sea anemone *Stichodactyla helianthus*, is a 35-residue disulfide-rich peptide that blocks the voltage-gated potassium channel Kv1.3 at *ca.* 10 pM and the related channel Kv1.1 at *ca.* 16 pM. We developed an analog of this peptide, ShK-186, which is currently in Phase 1b-2a clinical trials for the treatment of autoimmune diseases such as multiple sclerosis and rheumatoid arthritis. While ShK-186 displays a >100-fold improvement in selectivity for Kv1.3 over Kv1.1 compared with ShK, there is considerable interest in developing peptides with an even greater selectivity ratio. In this report, we describe several variants of ShK that incorporate *p*-phophono-phenylalanine at the *N*-terminus coupled with internal substitutions at Gln16 and Met21. In addition, we also explored the combinatorial effects of these internal substitutions with an alanine extension at the *C-*terminus. Their selectivity was determined by patch-clamp electrophysiology on Kv1.3 and Kv1.1 channels stably expressed in mouse fibroblasts. The peptides with an alanine extension blocked Kv1.3 at low pM concentrations and exhibited up to 2250-fold selectivity for Kv1.3 over Kv1.1. Analogs that incorporates p-phosphono-phenylalanine at the *N*-terminus blocked Kv1.3 with IC_50_s in the low pM range and did not affect Kv1.1 at concentrations up to 100 nM, displaying a selectivity enhancement of >10,000-fold for Kv1.3 over Kv1.1. Other potentially important Kv channels such as Kv1.4 and Kv1.6 were only partially blocked at 100 nM concentrations of each of the ShK analogs.

## 1. Introduction

Nature has provided us with an ancient pharmacopoeia of molecules with unique biological functions, which for many centuries, constituted the only therapeutics for treating disease. The diversity of life in the world’s oceans offers perhaps one of the greatest collections of natural products that could be exploited as potential therapeutic agents, yet this resource remains largely unexplored. With an estimated 250,000 marine animals and plants and potentially millions of marine microbes, the available chemical space of marine natural products is almost limitless.

The phylum Cnidaria includes some of the most venomous animals on our planet. Within this phylum, the Anthozoa class are found in both fresh water (hydroids) and salt water (sea anemones, jellyfish and corals) environments [1,2]. Most of these animals possess unique stinging cells known as cnidoblasts, which function like hypodermic needles, delivering a potent venom cocktail that helps to both immobilize prey and deter predation. As chromatographic separation methods have improved over the past several decades, the venom components from many of these species have been isolated and studied.

The venom of the Caribbean sun anemone, *Stichodactyla helianthus*, contains, among other components, large cytolytic proteins and numerous ion channel blocking peptides [3]. The cytolysins from this animal are approximately 17-kDa proteins with highly basic pI (>9), which cause hemolysis that can be inhibited by preincubation with sphingomyelin [4]. The first ion channel blocking peptide isolated was a Na^+^ channel blocker (ShN) composed of 48 residues with three disulfide bonds [5]. As separation procedures improved in the 1990’s, a minor venom component was isolated and characterized. This 35-residue peptide, ShK toxin, also contained three disulfide bonds but was the first peptide toxin from a sea anemone that blocked K^+^ channels [6].

**Figure 1:**
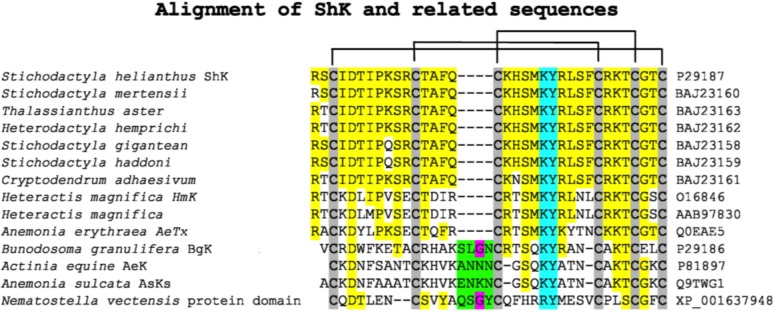
Sequence alignment of K^+^ channel blocking peptides from sea anemones. The GenBank accession codes are also indicated. Alignment of the sequences is via the Cysteine residues which are shaded in gray with the pairings indicated by the lines linking them. The yellow highlighted areas are indicating sequence identity. The cyan colored residues indicate the key pore blocking residues. The green region with the (conserved Gly residue in pink) represents a sequence insertion present in the BgK-type anemone toxins.

The chemical synthesis of ShK and further characterization identified a potent blocking activity of the peptide on Kv1.3 channels in Jurkat cells [7]. We began a systematic study of the peptide by mapping its disulfide bonds [8] and probing its K^+^ channel binding using alanine scanning [9,10,11]. Figure 1 shows the sequence of ShK and several other sea anemone-derived K^+^ channel blockers, as well as the location of their three intramolecular disulfide bonds. The high degree of homology in the peptides is highlighted in yellow. The Asp at position 5 is conserved across all of these peptides and has been shown to be critical for proper folding of the peptide as it forms a salt bridge with Lys30 [9,12,13]. The key K^+^ channel-binding residues are highlighted in cyan [9,10,11]; we found that the key residues in ShK for K^+^ channel blockade were Lys22 and Tyr23.

**Figure 2:**
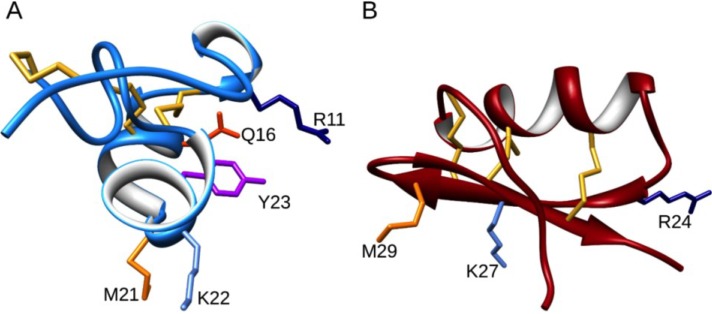
The solution structure of ShK in blue (PDB 1ROO) (**A**) compared with the scorpion toxin kaliotoxin in dark red (PDB 2UVS) (**B**). The side chains of critical residues are shown in stick format and colored navy blue (Arg), orange-red (Gln), magenta (Tyr), light-blue (Lys) and orange (Met).

We also determined the three-dimensional structure and solution properties of ShK using NMR spectroscopy [12,13]. Its solution structure is quite different from those of previously characterized K^+^ channel blocking peptides, such as the scorpion toxin kaliotoxin (Figure 2). As shown in Figure 2, ShK contains two small α-helical segments encompassing residues 14–19 and 21–24, with an extended *N*-terminal segment up to residue 8 and two interlocking turns that resemble a 3_10_ helix. In contrast, scorpion toxins have a dominant three-stranded antiparallel β-sheet with an α-helix lying across strands 2 and 3. Both ShK and kaliotoxin are stabilized by three disulfide bonds. Even though these peptides showed distinct secondary and tertiary structures, they blocked K^+^ channels in the same way, by occluding the pore region with a lysine residue. The position of the two key binding residues in ShK (Lys22 and Tyr23) is conserved in related K^+^ channel peptides from other sea anemones (Figure 1) [14]. The key ε-ammonium group of the Lys22 residue is located about 6.6 Å from the planar aromatic ring of the Tyr23 residue, a juxtaposition that led to the description of this pairing as a functional diad [14].

One of the striking aspects of ShK is its extremely potent blockade of voltage-gated K^+^ channels. In fact, it is one of the most potent blockers of Kv1.3 ever described, with an IC_50_ by patch-clamp of 10 pM [15] and equilibrium binding to Kv1.3 channel vesicles of 150 fM [16]. However, it also shows very potent blockade of other channel isotypes located in various important tissues, *viz.* Kv1.1 (cardiac), Kv1.4 (brain) and Kv1.6 (brain), each with pM affinity [17,18]. We have focused on developing analogs of ShK that would improve its selectivity profile and ultimately enhance its potential as a therapeutic lead.

The Kv1.3 channel was discovered in the early 1980’s by the Chandy and Cahalan labs at the University of California Irvine [19] and the Deutsch lab at the University of Pennsylvania [20]. This K^+^ channel, found in T lymphocytes, was the first to be described outside electrically excitable tissues. Kv1.3 channel research over the next two decades helped to define its role in autoimmune diseases and show that it was an important target for pharmaceutical development [17,18,21]. Kv1.3 in T lymphocytes is a voltage-gated homotetrameric membrane protein responsible for controlling the membrane potential when these cells are terminally differentiated into effector memory T cells (T_EM_ cells) [17,18,21]. Studies on T_EM_ cells isolated from patients with chronic inflammatory diseases clearly showed that these cells are responsive to antigens known to be implicated in diseases such as multiple sclerosis, rheumatoid arthritis, type-1 diabetes and asthma, and have greatly elevated numbers of Kv1.3 channels in these conditions [22,23,24,25]. Furthermore, medical reports of people with autoimmune diseases documented amelioration of their symptoms following scorpion envenomation, prompting interest in understanding the cause of this effect [26]. With numerous scorpion venom peptides already isolated and characterized, kaliotoxin was showed to ameliorate clinical signs of acute adoptive experimental autoimmune encephalomyelitis (EAE), an animal model of MS mediated by T lymphocytes [27]. As a continuation of this work, we demonstrated that both ShK and its analog ShK-170 were efficacious in reducing severity in acute adoptive EAE and in preventing a delayed type hypersensitivity reaction [28,29].

We have improved the drugability of ShK by making more stable and Kv1.3-selective variants of the peptide. Our lead peptide, ShK-186 (dalazatide, the FDA approved name), has improved stability through amidating the *C*-terminus and presenting the selectivity-enhancing phosphotyrosine residue at the *N*-terminus. This peptide has a >100-fold improvement in selectivity for Kv1.3 over Kv1.1 and >1000-fold over Kv1.6, and shows efficacy in a number of animal models of chronic inflammatory diseases [22,23,30,31]. Other non-hydrolysable analogs of the *N*-terminal pTyr, such as para phosphonphenylalanine (Ppa), have also been prepared, with and without substitutions of Met21 (ShK-190) with the nonoxidizable Nle residue (ShK-192) [28,32]. These peptides had improved stability profiles relative to ShK-186 and ShK-170 and demonstrated similar selectivity profiles to ShK-186. ShK-186 was well tolerated in Phase 1a and 1b clinical trials in healthy volunteers and has been advanced by Kineta Inc. (Seattle, WA, USA) into Phase 1b-2a clinical trials with patients [33].

We have continued to develop ShK analogs with even greater Kv1.3 selectivity ratios [34,35]. In this report, we describe new analogs that incorporated the Q16K substitution into our ShK-190 and 192 templates, which contained the *N*-terminal Ppa and an amidated *C*-terminus, both with and without the Met21 to Nle substitution. In addition, we generated recombinant analogs to probe the importance of the Q16K substitution in combination with an additional *C*-terminal Ala residue and the substitution M21I.

## 2. Results and Discussion

### 2.1. Synthesis, Expression and Folding

**Figure 3:**
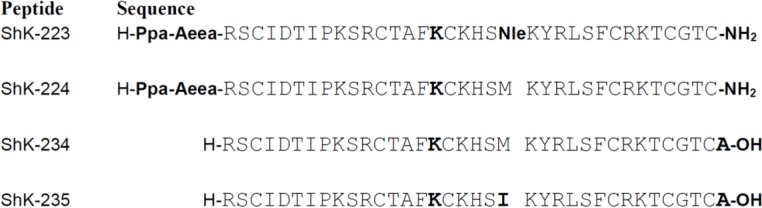
Sequences of ShK analogs described in this study.

A series of analogs was designed to probe the Q16K substitution along with other selectivity determinants. ShK-223 and ShK-224 (Figure 3) were designed to include a substitution that a group at Amgen had published in a patent filed in 2007; this substitution replaced Gln16 with Lys [36]. The results presented in that patent suggested that this substitution conferred on ShK a high Kv1.3 *versus* Kv1.1 specificity. Thus, we incorporated this substitution into ShK-192, which incorporated a non-hydrolyzable para-phosphono-Phe as the *N*-terminal residue, extended from the primary ShK sequence with an Aeea (aminoethyloxyethyloxyacetyl, mini-Peg™) linker, as well as amidation at the *C*-terminus (ShK-224). We also incorporated a Met21 to Nle substitution into this sequence to generate an analog that would be less susceptible to oxidation (ShK-223). Additionally, we recombinantly expressed two additional analogs, both with *C*-terminal Ala additions and the Q16K substitution (ShK-234), and one with Met21 replaced with Ile (ShK-235).

**Figure 4:**
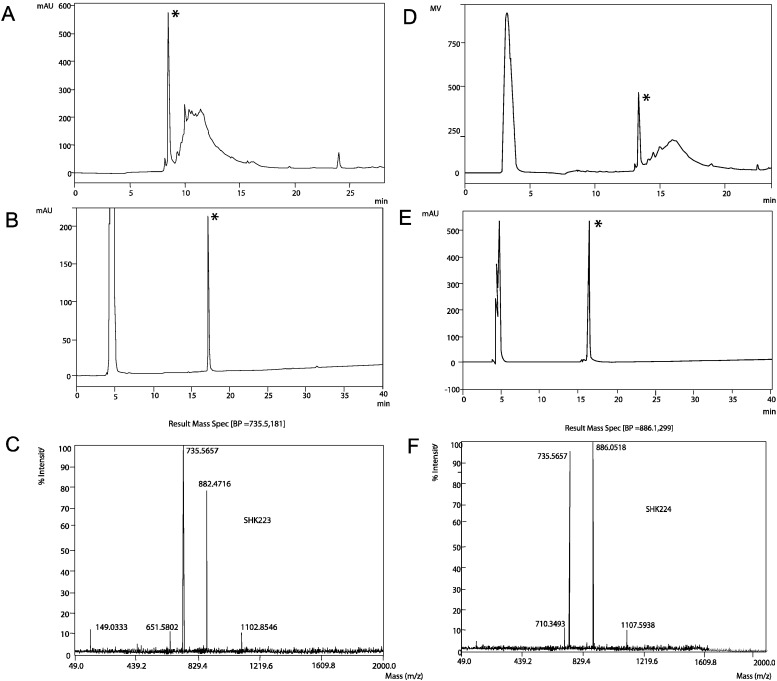
(**A**) RP-HPLC analysis of oxidative folding of ShK-223 after overnight folding (gradient conditions 10%–70% B in 30 min, 1 mL/min, A^220^ (absorbance at 220 nm), 0.1 AUFS (absorbance full scale) (**B**) RP-HPLC analysis of the purified ShK-223; (**C**) ESI-MS analysis of the multi-charged ions from ShK-223; (**D**) RP-HPLC analysis of oxidative folding of ShK-224 after overnight folding; (**E**) RP-HPLC analysis of the purified ShK-224; (**F**) ESI-MS analysis of the multi-charged ions from ShK-224. (Asterisk indicates correctly folded ShK peptides).

ShK-223 and ShK-224 were synthesized on a Rink-mBHA resin using an Fmoc-tBu strategy. The solid-phase assembly proceeded smoothly with all couplings mediated by 6-Cl-HOBT and diisopropylcarbodiimide. In order to form the disulfide bonds utilizing a simple redox buffer, all Cys residues were protected with the trityl group. Following synthesis of the primary chain, each peptide was cleaved from the resin and simultaneously deprotected using an acidolytic reagent cocktail containing carbocation scavengers. The peptides were folded to the active form using a slightly basic aqueous buffer containing an equimolar ratio of reduced and oxidized glutathione. The peptide folding was allowed to proceed 16 h and was determined to be complete with the formation of the major front eluting peak consistent with other ShK peptides as shown in Figure 4. Each peptide was purified by RP-HPLC and the purified peptides were characterized by analytical HPLC and ESI-MS, as shown in Figure 4. Each HPLC chromatogram shows an injection peak at approximately 5 min which includes buffer salts whereas the folded peptide elution peak is observed at approximately 16–17 min in the gradient employed in Figure 4B,E.

ShK-234 and ShK-235 were designed on the basis of data presented in the Amgen patent [36]. When the Q16K mutation was combined with an alanine extension at the *C*-terminus and/or a hydrophobic substitution at position 21, significant enhancement of selectivity for Kv1.3 over Kv1.1 was observed. In addition, an alanine extension produced greater improvement in Kv1.3-selectivity than extension with Val, Glu or Tyr. ShK-234 incorporated the Q16K substitution and Ala extension, while ShK-235 also incorporated a M21I substitution in order to eliminate a potential site of oxidation. Both of these analogs were expressed recombinantly as fusion proteins with thioredoxin and hexa-histidine tags. The resulting inclusion bodies were solubilized using buffers containing denaturants followed by on-column refolding by gradual removal of denaturants, as described previously [37]. Both analogs were cleaved with sequence-specific protease enterokinase to release the tags. The analogs were purified to homogeneity by RP-HPLC with an optimized acetonitrile gradient.

### 2.2. Efficacy and Selectivity of ShK Analogs on K^+^ Channels

**Figure 5:**
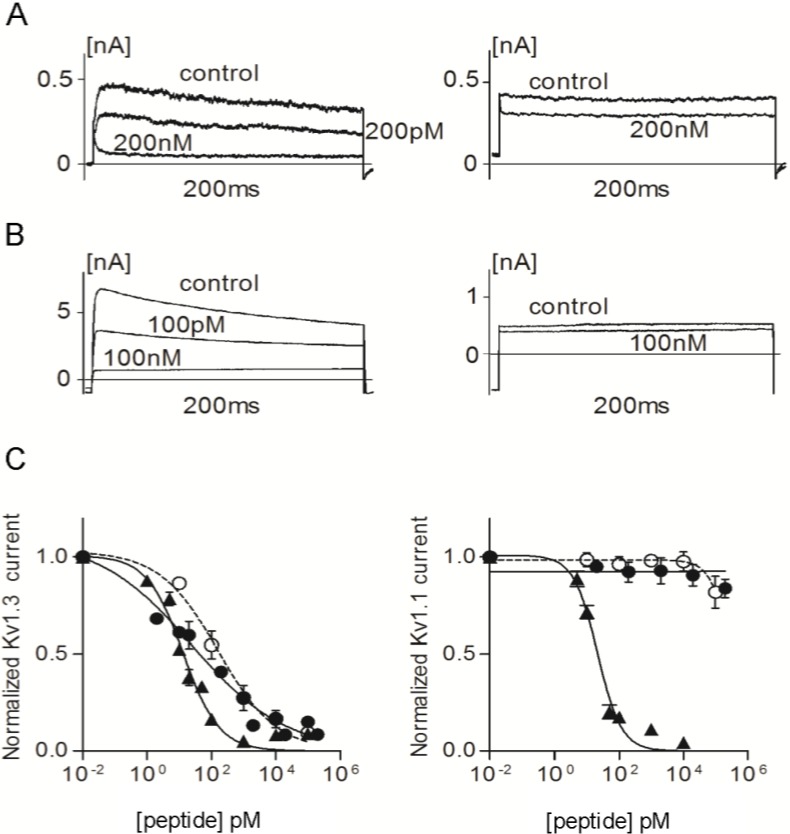
(**A**) Whole-cell Kv1.3 (**left**) and Kv1.1 (**right**) currents before (control) and after addition of ShK-223; (**B**) Whole-cell Kv1.3 (**left**) and Kv1.1 (**right**) currents before (control) and after addition of ShK-224; (**C**) Dose-response effects of ShK (▲), ShK-223 (●), and ShK-224 (○) on Kv1.3 (**left**) and Kv1.1 (**right**) currents (*n* = 3 to 6 cells per concentration).

**Figure 6:**
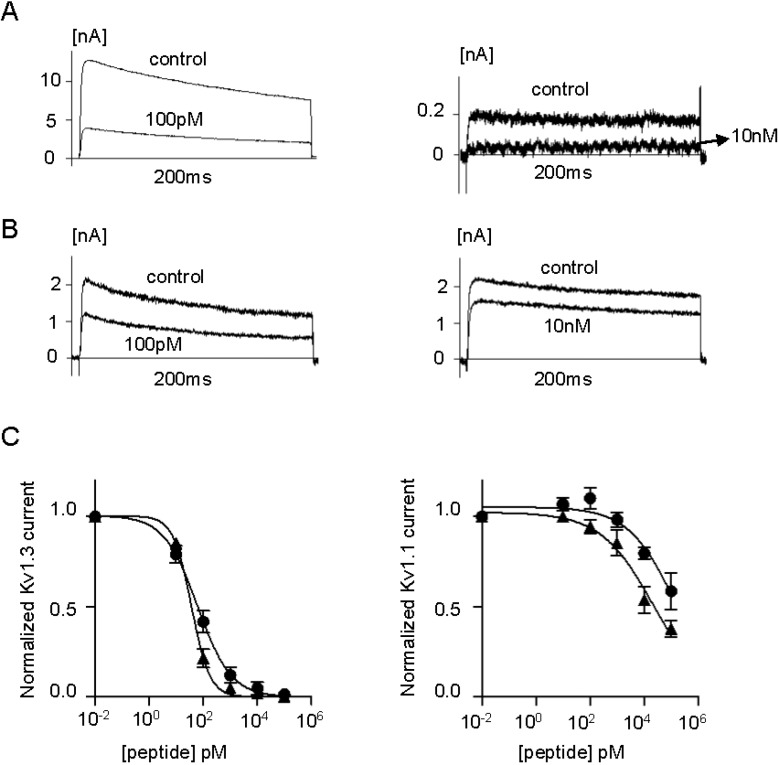
(**A**) Whole-cell Kv1.3 (**left**) and Kv1.1 (**right**) currents before (control) and after addition of ShK-234; (**B**) Whole-cell Kv1.3 (**left**) and Kv1.1 (**right**) currents before (control) and after addition of ShK-235; (**C**) Dose-response effects of ShK-234 (▲) and ShK-235 (●) on Kv1.3 (**left**) and Kv1.1 (**right**) currents (*n* = 3 to 4 cells per concentration).

We used patch-clamp electrophysiology to assess the effects of ShK-223, ShK-224, ShK-234 and ShK-235 on Kv1.1 and Kv1.3 channels and compare them to the parent peptide, ShK. Mouse fibroblasts stably expressing homotetramers of Kv1.1 or Kv1.3 were patch-clamped in the whole-cell configuration and steady-state block was measured after addition of different concentrations of the peptides. As published previously, ShK inhibited Kv1.3 currents with an IC_50_ of 13.3 ± 1.40 pM and Kv1.1 currents with an IC_50_ of 21.5 ± 2.26 pM (Figure 5, Table 1) [15,36]. ShK-224 exhibited a 12-fold loss of efficacy on Kv1.3 relative to ShK, ShK-235 a 5-fold loss, and ShK-234 a 2-fold loss, whereas the potency of ShK-223 on Kv1.3 was similar to that of ShK (Figure 5 and Figure 6, Table 1). In contrast to ShK, ShK-234 exhibited a 580-fold selectivity for Kv1.3 over Kv1.1, and ShK-235 a 2250-fold selectivity, while neither ShK-223 nor ShK-224 had any effect on Kv1.1 currents at concentrations up to 100 nM, representing a selectivity for Kv1.3 of >10,000-fold.

**Table 1:** Selectivity of ShK and its analogs determined by whole-cell patch-clamp electrophysiology.

Channel	Kv1.1	Kv1.3	Kv1.4	Kv1.6
ShK	IC_50_ = 21.5 ± 2.2 pM	IC_50_ = 13.3 ± 1.4 pM	IC_50_ = 312 ± 14 pM	IC_50_ = 165 ± 8 nM
ShK-223	<10% block at 100 nM	IC_50_ = 25 ± 14 pM	<5% block at 100 nM	<10% block at 100 nM
ShK-224	<10% block at 100 nM	IC_50_ = 164 ± 59 pM	<20% block at 100 nM	~25% block at 100 nM
ShK-234	IC_50_ = 21.5 ± 8 nM	IC_50_ = 37 ± 4.6 pM	<15% block at 100 nM	~25% block at 100 nM
ShK-235	IC_50_ = 140 ± 62 nM	IC_50_ = 62 ± 13.2 pM	<15% block at 100 nM	~25% block at 100 nM

We also tested the effects of ShK-223, ShK-224, ShK-234 and ShK-235 on Kv1.4 (mouse LTK fibroblasts stably transfected with Kv1.4, a kind gift from Dr. Michael Tamkun, Colorado State University) and Kv1.6 (HEK cells transiently transfected with hKv1.6:eGFP 2:1, a gift from Dr. Heike Wulff, University of California, Davis) using Lipofectamine 2000. The HEK cells were patched 19 h after transfection. With each analog tested on Kv1.4 and Kv1.6, only a partial block was observed at a peptide concentration of 100 nM (Table 1).

Previously, we have shown that *N*-terminal extension of ShK with a negatively-charged moiety such as phospho-Tyr of Ppa enhanced its selectivity for Kv1.3 over Kv1.1 and all other tested channels by >100-fold [32]. In addition, modification of the *C*-terminus of ShK by addition of a Lys and *C*-terminal amidation enhanced selectivity by 36-fold, although *C*-terminal amidation of ShK itself gave rise to equal potency against both channels [34]. Unpublished results reported in an Amgen patent [36] suggested that replacement of Gln16 by Lys and extension of the *C*-terminus with an Ala independently enhanced selectivity for Kv1.3. In this study, we have combined several of these modifications with replacement of Met21 by hydrophobic residues (Nle, Ile) to generate potent and selective blockers of Kv1.3. We have not yet constructed models of these analogs docked with Kv1.3 and Kv1.1 to gain insight into the molecular basis of the improved selectivity although our recent studies [34,35,38,39] suggest that this would be informative and may identify additional modifications to further enhance selectivity.

## 3. Experimental Section

### 3.1. Peptide Synthesis

Boc-Ppa(tBu)_2_-OH was synthesized as reported previously [34]. ShK (Q16K)-amide peptide was synthesized on a Prelude peptide synthesizer using Fmoc-Rink-amide resin (Peptides International, Louisville, KY, USA) and a standard Fmoc-tBu protecting group strategy. All Cys residues were Trityl protected. All couplings were mediated with diisopropylcarbodiimide (DIC) with 6-Cl-HOBT. Prior to coupling the Met at position 21, the resin was split into two portions. One portion was coupled with Met and the second with Nle. Synthesis then continued until completion of the 35-residue parent sequence, with Gln16 being substituted with Lys. At this point the linker Fmoc-Mini-Peg™ linker (Fmoc-aminoethyloxyethyloxyacetic acid; Aeea) was coupled to each product using the same DIC-6-Cl-HOBT chemistry followed by the *N*-terminal Boc-Ppa(tBu)_2_-OH. Following primary assembly of the 37-residue peptides, the crude peptides were deprotected using a trifluoroacetic acid cocktail containing H_2_O, triisopropylsilane, anisole, and thioanisole (9:0.2:0.2:0.2:0.2) for 2 h at room temperature. The crude linear products were obtained by ether precipitation following acidolytic cleavage then dissolved in 50% aqueous acetic acid and diluted into water to a concentration of 0.2 mg/mL. The pH of this peptide solution was adjusted to 8.0 with NH_4_OH, and reduced and oxidized glutathione were added to a final concentration of 0.5 mM. Following overnight oxidative folding, the peptides were purified by preparative RP-HPLC using a 5 cm × 45 cm preparative column packed with Luna ODS, 10 μ, 120 Å with a linear gradient of H_2_O containing 0.05% TFA *versus* acetonitrile from 5% to 35% MeCN in 75 min at a flow rate of 100 mL/min. Fractions were collected by monitoring the eluate at 225 nm. Fractions with a purity >95% were pooled and lyophilized. The final products were analyzed by RP-HPLC for final purity and ESI-MS using an Applied Biosystems Mariner Electrospray mass spectrometer (Applied Biosystems, Farmingham, MA, USA).

### 3.2. Recombinant Peptide Production

The expression and purification of ShK have been described previously [37]. Briefly, competent *E. coli* BL21(DE3) cells were transformed with pET32a plasmid containing ShK-234 and ShK-235 nucleotide sequences. Cells were cultured overnight at 37 °C in Luria-Bertani (LB) medium and added to 1 L LB broth, which was cultured at 37 °C until the optical density at 600 nm (OD_600_) reached 0.5–0.8. The culture was equilibrated at 28 °C for 1 h prior to induction by isopropyl-β-d-thiogalactoside (IPTG) at a final concentration of 1 mM. The cells were then spun down and lysed with bugbuster master mix (Novagen, Madison, WI, USA) with the addition of the EDTA free protease inhibitor (Roche, Indianapolis, IN, USA). The inclusion bodies were collected by centrifugation and solubilized with denaturing buffer followed by refolding on Ni-NTA column by gradual reduction of denaturant concentrations, as reported previously [37]. The refolded recombinant proteins were then cleaved with enterokinase (New England Biolabs, Ipswich, MA, USA) and purified to homogeneity by RP-HPLC using a C18 column (Phenomenex, 100 × 10.0 mm). The eluted fractions containing ShK analogs were lyophilized for storage.

### 3.3. Patch-Clamp Electrophysiology

The effects of ShK and its new analogs were tested on mKv1.1 and mKv1.3 channels stably expressed in mouse L929 fibroblasts (gifts from Dr. K. George Chandy, University of California, Irvine, CA, USA) in the whole-cell configuration of the patch-clamp technique on a Port-a-Patch (Nanion Technologies, North Brunswick, NJ, USA) connected to an EPC10-USB amplifier (HEKA Instruments, Bellmore, NY, USA) as described [35,36]. Chips averaged 2–3.5 MΩ and a holding potential of −80 mV was used for all recordings. Series resistance compensation of 80% was used when currents exceeded 2 nA. The external solution was normal Ringer solution containing (in mM): 160 NaCl, 4.5 KCl, 2 CaCl_2_, 1 MgCl_2_, 10 Hepes, pH 7.4, 310 mOsm. The internal solution contained (in mM): 145 KF, 10 HEPES, 10 EGTA (ethylene glycol tetraacetic acid), 2 MgCl_2_, pH 7.2, 300 mOsm. Currents were elicited by repeated 200 ms pulses to 40 mV applied at intervals of 30 s for Kv1.3 and 10 s for Kv1.1. IC_50_ values were determined by fitting the Hill equation to the reduction of area under the current curve measured at 40 mV in the presence of different concentrations of the peptides (GraphPad Prism software, La Jolla, CA, USA).

## 4. Conclusions

We have synthesized two analogs of ShK containing the substitution Q16K coupled with the *N*-terminal Ppa, *C*-terminal amidation, and either the native Met or a hydrophobic analogue, Nle. The synthesis of these products was similar to many other ShK analogs, which would facilitate chemical manufacture of these as viable potential drug candidates. The *N*-terminal extension Ppa in combination with the oxidation-stable Nle21 (ShK-223) had greatly enhanced selectivity, with increased potency on Kv1.3 relative to the natural Met (ShK-224). Recombinant *C*-terminal acid peptides incorporating the Q16K substitution and a *C*-terminal Ala extension (ShK-234) also showed significant selectivity enhancement. A second recombinant analog with *C*-terminal Ala, Q16K and Met21 replaced by Ile (ShK-235) showed an even greater selectivity for Kv1.3 over Kv1.1. These peptides would be amenable to registration as biologics as they can be produced by recombinant expression. These four peptides demonstrated selectivity for Kv1.3 over Kv1.1 ranging from 580-fold (ShK-234) to more than a 10,000-fold (ShK-223) with very little block of other important Kv subtypes (Kv1.4 and Kv1.6) at a concentration of 100 nM. The enhanced selectivity of these four analogs for Kv1.3 makes them attractive potential second-generation leads for ShK-186, which is currently in Phase 1b-2a clinical trials.
